# Antagonistic Potential of Soil *Streptomyces* Isolates from Southern Thailand to Inhibit Foodborne Bacterial Pathogens

**DOI:** 10.1155/2021/2545441

**Published:** 2021-08-30

**Authors:** Waenurama Chemoh, Wahida Bin-Ismail, Sawitree Dueramae

**Affiliations:** ^1^Department of Microbiology, Faculty of Medicine, Princess of Naradhiwas University, Narathiwat 96000, Thailand; ^2^Faculty of Science and Technology, Princess of Naradhiwas University, Narathiwat 96000, Thailand; ^3^Department of Applied Microbiology, Institute of Food Research and Product Development, Kasetsart University, Bangkok 10900, Thailand

## Abstract

*Streptomyces* are well known for their competence to produce thousands of bioactive secondary metabolites and enzymes. This study aimed to assess the inhibitory activities of crude extracts from diverse *Streptomyces* collected from rice soils in Narathiwat, Thailand, against foodborne bacterial pathogens. In total, 136 *Actinomycete* isolates were screened using a cross-streak method for the ability to produce effective metabolites against 5 pathogenic bacteria. Out of these, 19 (13.97%) isolates had antibacterial activity against at least one tested bacterium. Most of the isolates could strongly suppress the growth of *S. aureus* ATCC25923 and *B. cereus* MTCC430 except *P. aeruginosa* ATCC27853. On the basis of morphological, cultural, and biochemical characteristics, all potent isolates exhibited typical features that fitted the genus *Streptomyces*. Two of the 7 selected ethyl acetate crude extracts had good antagonistic activity against *S. aureus* ATCC25923 and *B. cereus* MTCC430 when tested using the agar well diffusion assay. Furthermore, minimum inhibitory concentration (MIC) and minimum bactericidal concentration (MBC) values of the 2 extracts evaluated using the colorimetric broth microdilution method ranged from 256 to >1,024 *μ*g/ml against the tested bacteria. The partial nucleotide sequences of the *16S rRNA* gene led to identifying both active isolates as *Streptomyces* species. These active *Streptomyces* isolates could provide an interesting source for generating innumerable natural compounds with antibacterial activity that can presumably be developed to fight bacterial pathogens in the near future.

## 1. Introduction

Foodborne diseases are a vital health public concern that annually affect one-third of people worldwide. WHO reported that the burden of foodborne illness in low-income countries is much higher than in high-income countries [1]. In addition, the WHO reported the highest occurrence of both foodborne diseases and fatality rate in the African and Southeast Asian regions. Global foodborne infection is responsible for 230,000 deaths from 550 million people contracting a foodborne illness from diarrheal diseases yearly, which accounts for 30% of all deaths in children aged below five years [1].

*Streptomyces* are well known for their competence to produce thousands of bioactive secondary metabolites and enzymes [2, 3]. The genus consists of filamentous Gram-positive bacteria belonging to the phylum *Actinobacteria*. The species can grow in a variety of habitats such as soil, marine sediments, and marine sponges [4, 5]. Copious isolates of *Streptomyces* have been used to make the majority of natural-derived antibiotics [6]. Consequently, they are regarded as the largest group of microorganisms with medicinal applications, generating more than 50% of clinically useful antibiotics with antibacterial, antifungal, antiviral, anthelmintic drugs and immune modulators [7, 8]. Other relevant secondary metabolites produced by *Streptomyces* also exhibit many kinds of biological activities and are used as pharmacological agents and agrobiologicals [9, 10].

The discovery of natural, bioactive compounds with antimicrobial activity may result in the development of novel drugs of choice for treating patients infected with bacterial diseases. Annually, bacterial infections are involved in about 17 million deaths (mostly children and the elderly), while infectious diseases are still the second leading cause of death globally [11]. However, the speedy emergence of resistant bacteria is currently a major public health issue in many parts of the world, especially in developing countries [12]. The outcomes of antibiotic resistance can invoke greater risks of worse clinical consequences for patients, more expensive medical costs, and increased death rates [13–15]. Recent studies have shown the most common, infamous, antibiotic-resistant bacteria which have caused infections in hospitals and communities include *Staphylococcus aureus, Enterococcus faecium, Klebsiella pneumoniae, Acinetobacter baumannii, Pseudomonas aeruginosa*, and *Enterobacter* species [16]. People are generally exposed to these drug-resistant bacteria via farm animals, aquaculture, and food handlers [17]. Antibiotic resistance often develops due to overuse or misuse of antibiotics and a lack of awareness that this directly promotes the ability of the pathogens to adapt to several common antibiotics. The prevalence of multidrug-resistant (MDR) bacterial infections reported from nine provincial hospitals in Northeast Thailand has been recorded as 3–75% [18], and most of the patients acquired opportunistic infection from the hospitals. Thus, there is an urgent demand for new antibiotics to combat these bacteria. Therefore, this study aimed to isolate *Streptomyces* from various soil samples in Narathiwat, Southern Thailand, with potent antibacterial activities against pathogenic bacteria. The results of this findings may lead to providing a potential source of new bioactive secondary metabolites.

## 2. Materials and Methods

### 2.1. Sample Collection and Isolation of *Actinomycetes*

**Figure 1:**
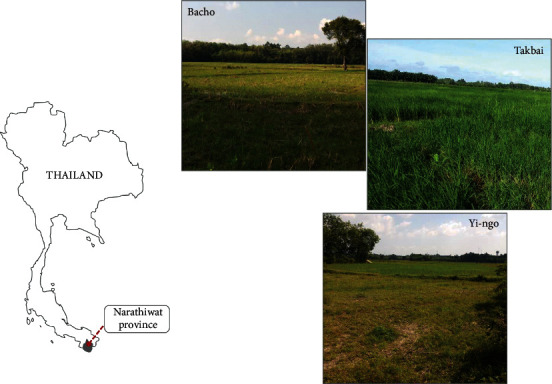
Map and some paddy fields from sampling areas in Bacho, Tak Bai, and Yi-ngo districts.

Thirty soil samples were collected from 3 different sites located in Narathiwat Province, Thailand, in Bacho, Tak Bai, and Yi-ngo districts (Figure 1). The samples were randomly taken from paddy field (10 fields per one area). A trowel was used to delve the soil. Approximately, 100 g of each soil sample was taken about 10 cm depth after removing about 3 cm of the soil surface. These samples were placed in sterile plastic bags, closed tightly, and transferred immediately to the laboratory. Some physicochemical parameters of the soil including texture, color, pH, and moisture content were determined [19, 20]. Later, the samples were air-dried at 45°C for 4 h, and subsequently crushed, and sieved. The soil was applied for pretreatment process with drying at 70°C for 1 h in hot air oven. The serial dilution method using preheated soil provided *Actinomyces* isolates. Briefly, 1 g of dried soil was suspended in 9 ml sterile distilled water and serially diluted in sterile water up to 10^−5^. An aliquot of 0.1 ml of each dilution was spread on a plate of starch casein agar medium (SCA, Himedia, India) supplemented with 25 *μ*g/ml of nalidixic acid (Sigma-Aldrich, USA) and 50 *μ*g/ml of amphotericin B (Sigma-Aldrich, USA) to restrain Gram-negative bacterial and fungal contaminations, respectively. Plates were incubated at 30°C for 7–14 days. After the incubation period, each plate was observed for the presence of *Actinomycetes* colonies that typically have a powdery or adhesive character, with creased or dry features and branching filaments with or without aerial mycelia [21]. The colonies of *Actinomyces* were re-streaked on yeast extract malt extract agar medium (ISP-2, Himedia, India) and incubated at 30°C for 7–14 days. The pure *Actinomyces* colonies were individually preserved on an agar slant of ISP-2 at 4°C and maintained in 25% (v/v) glycerol stocks before being stored at −80°C until used.

### 2.2. Screening of Antibacterial Activity

The antibacterial activity of the *Actinomyces* isolates was examined by primary screening against the following 5 selected human pathogenic bacteria: *Escherichia coli* ATCC25922, *Salmonella enterica* serovar Typhimurium ATCC14028, *Pseudomonas aeruginosa* ATCC27853*, Bacillus cereus* MTCC430, and *Staphylococcus aureus* ATCC25923. The test microorganisms were inoculated in tryptic soy broth (TSB, Himedia, India) at 37°C for 18–24 h. The antagonistic assay was initially screened using the cross-streak method [22]. Each isolate of *Actinomyces* was inoculated on ISP-2 at 30°C for 7–14 days. It was then re-streaked on Mueller Hinton agar (MHA, Himedia, India) as a direct line in the center of the plate and incubated at 30°C for 7 days. Each test pathogenic bacterium was cultured using a streak line perpendicular to the *Actinomyces* isolates on the same MHA plate and incubated at 35°C for 24 h. The inhibitory effect of the *Actinomyces* isolates against the growth of the tested strains was evaluated using the detection of the inhibition zone, and the results were indicated as follows: − = no activity; + = weak activity (<50% inhibition); ++ = moderate activity (50–80% inhibition); and +++ = strong activity (>80% inhibition).

### 2.3. Characterization and Identification of Presumptive *Streptomyces* Isolates

The effective *Actinomycetes* obtained from the antibacterial screening were characterized for the presence of *Streptomyces* using a morphological method, which mainly relied on Gram staining, aerial mass color, and spore formation. Morphological observation was determined by the growth on ISP-2 agar incubated at 30°C for 7–14 days and then by microscopic examination of Gram-stained slides. The colors of the aerial mycelia produced by presumptive *Streptomyces* isolates were compared with the National Bureau of Standards Color Chart [23]. The spore chain arrangement was inspected under oil immersion (1,000x) using a slide culture method. In addition, identification of the *Streptomyces* isolates was based on 7 biochemical tests: catalase reaction, urea hydrolysis, H_2_S production, indole production, citrate degradation, the motility test, and the lysine decarboxylase (LDC) test. The *Streptomyces* isolates were identified to the genus level according to Bergey's Manual of Systematic Bacteriology [24].

### 2.4. Preparation of Crude Extracts

The extraction of active compound was performed following Taechowisan et al. [25] with minor modification. Forty-five ml of ISP-2 broth was inoculated with 5 ml of spore suspension (1 × 10^8^ spores/ml) of the 10-day-old selected presumptive *Streptomyces* strains grown on ISP-4 (Himedia, India) plates and incubated at 30°C with agitation at 120 rpm for 2 days. After incubation, 50 ml of this culture was aseptically transferred into 450 ml of ISP-2 broth and then incubated for 5 days under the same conditions. The culture broth was filtered through Whatman filter paper to collect the supernatant. Ethyl acetate (J.T.Baker, USA) was added to the supernatant at a ratio of 1 : 1 (v/v) and shaken vigorously for 2 days. The solvent phase was vaporized to dryness at 50°C using a rotary evaporator (Heidolph, Germany). The filtrate of ethyl acetated crude extract was dissolved in 5 ml of acetone, dried, and kept at 4°C for further assay.

### 2.5. Preliminary Test for Bioactive Metabolites of Ethyl Acetate Crude Extracts

The inhibitory effect of the collected crude extracts on the tested bacteria was determined using the agar well diffusion method with slight modifications [26]. Briefly, the bacteria were grown in TSB at 35°C for 18–24 h, after which the cell pellet was harvested using centrifugation at 2,000 ×g for 10 min. The pellet was resuspended in 0.85% normal saline to a final cell density of 0.5 McFarland standards and then was swabbed on the top of MHA plates. A sterile 6 mm cork borer was used to punch holes in the inoculated plates. The 10 mg/ml stock extract solution was prepared by dissolving the dry crude extract in 10% dimethyl sulfoxide (DMSO, Merck, United States), and thereafter 50 *μ*l of each extract was loaded into the holes. The plates were left for 45 min at room temperature until the extract was completely dispersed into the medium before incubation at 35°C for 18–24 h. Each experiment was done in triplicate. The 1 mg/ml streptomycin (Sigma-Aldrich, USA) and 10% DMSO served as positive and negative controls, respectively. The diameter of a clear zone surrounding the well was measured in millimeters and the results were later reported as the mean ± standard deviation of 3 repeats. The presumptive *Streptomyces* isolates showing more potent antibacterial activities were further selected to determine the minimum inhibitory concentration (MIC) and minimum bactericidal concentration (MBC) of partly purified extracellular crude extracts on the test pathogens.

### 2.6. Determination of MIC and MBC of Crude Extracts

The MIC and MBC of the ethyl acetate crude extract were evaluated using the colorimetric broth microdilution method with some modifications [27]. The extract was reconstituted in 10% DMSO solution with distilled water to finally obtain a concentration of 2,048 *μ*g/ml. Then, a 2-fold dilution series of the extract was made with double strength MHB in 96-well, flat-bottomed microtiter plates to eventual concentrations varying from 1 to 1,024 *μ*g/ml. An aliquot (50 *μ*l of 1 × 10^6^ CFU/ml) of tested bacterial suspension was subsequently introduced into the wells. The plate was incubated at 35°C for 15 h. The positive control streptomycin (100 *μ*g/ml) and sterility control (0.85% NaCl) were included in each test. All assays were conducted in triplicate against the tested inoculum. After the incubation period, 50 *μ*l of 0.2 mg/ml solution of 2,3,5-triphenyl tetrazolium chloride (Himedia, India) was loaded in all wells to affirm the growth of bacteria and further incubated at 35°C for 3 h. The alteration of the color in the well to pink indicated the presence of bacterial growth, while a lack of any pink color at the lowest concentration of the extract was regarded as the MIC value. In addition, one loop from each dilution that exhibited the growth inhibition was streaked on a TSA plate to note the survival of the pathogens. The concentration of extract resulting in no growth of bacterial colonies on the plate was considered as the MBC value. The degree of inhibition for the MIC determination of extracts was classified based on the following: strong inhibitor, MIC of <512 *μ*g/ml; moderate inhibitor, MIC of 512–1,024 *μ*g/ml; and weak inhibitor, MIC of >1,024 *μ*g/ml [28].

### 2.7. Molecular Identification and Sequencing of the *16S rRNA* Gene

The selected *Streptomyces* isolates showing the strong antagonistic activity against tested bacteria were cultured in 1 ml of nutrient broth at 30°C for 96 h with agitation. The pellets were collected using centrifugation at 6,000 ×g for 10 min. The genomic DNA extraction was conducted using QIAamp DNA mini kits (Qiagen, Germany) following the manufacturer's guidance for isolation of genomic DNA from Gram-positive bacteria. PCR amplification of the *16S rRNA* gene was performed using universal primers 27F and 1492R [29]. The 1,400-bp 16S rRNA amplicons were purified using a GenepHlow Gel/PCR kit (Geneaid, Taiwan) and were subsequently sequenced (First Base Company, Malaysia) in both forward and reverse directions with the same primers as above. The purified sequences were compared with sequence data deposited in the GenBank database using the NCBI BLAST package.

### 2.8. Data Analysis

Statistical data analysis was carried out using the SPSS version 26 software package (IBM Corp., USA). The mean comparisons of inhibition zone diameters between the crude extracts and the test pathogens were evaluated using a one-way ANOVA test with Dunnett T3 as post hoc analysis. Statistical outcomes with *P* < 0.05 were considered statistically significant.

## 3. Results

### 3.1. Sample Collection and Isolation of *Actinomycetes*

The data of physicochemical properties of the 30 soil samples collected from three locations in Narathiwat are presented in Table 1. The distribution of *Actinomycete* isolates was found in those samples. In total, 136 *Actinomycete* isolates were grown on SCA medium (Table 1), of which 45 (33.09%), 57 (41.91%), and 34 (25.00%) *Actinomycetes* were isolated from Bacho, Tak Bai, and Yi-ngo soil samples, respectively.

### 3.2. Screening of Antibacterial Activity

All 136 *Actinomyces* isolates were screened for antagonistic activity against 5 selected bacterial pathogens. Among them, only 19 isolates (13.97%) were active isolates (Table 2) and of these, 14 isolates (73.68%) could strongly inhibit the growth of *B. cereus* MTCC430, while 12 isolates (63.16%) had the strongest inhibition against *S. aureus* ATCC25923, 8 isolates weakly or moderately suppressed the growth of *E. coli* ATCC25922, and 2 isolates had a weak inhibitory effect on the growth of *S. enterica* serovar Typhimurium ATCC14028. None of the isolates could inhibit the growth of *P. aeruginosa* ATCC27853. The most sensitive bacteria to metabolites produced by these *Actinomycetes* were *B. cereus* MTCC430 and *S. aureus* ATCC25923; consequently, they were selected for further testing.

### 3.3. Characterization and Presumptive Identification of *Streptomyces* Isolates

The 19 active *Actinomyces* isolates were primarily classified as *Streptomyces* based on their morphological appearance including colors of aerial mycelium and the presence of spore formation. Four colors of aerial mycelium were identified, with most isolates being gray (9 isolates; 47.37%), white (6 isolates; 31.58%), or yellow (3 isolates; 15.79%) as shown in Table 3. The slide cultures revealed diverse shapes of spore chains depending on each isolate with spiral, rectiflexibile, and retinaculiaperti; however, most isolates (42.11%) had retinaculiaperti spore chains. The biochemical characteristics of all isolates are shown in Table 3. These isolates were Gram-positive and produced catalase. No isolates produced indole or H_2_S. The degradation of citrate and urea was noted in some isolates. All isolates were non-motile and negative for the LDC test.

### 3.4. Preliminary Test for Bioactive Metabolites of Ethyl Acetate Crude Extracts

**Figure 2:**
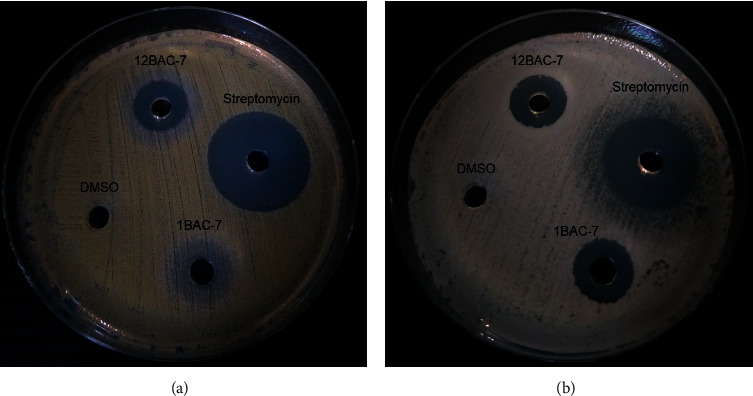
Inhibitory effect of 1BAC-7FE and 12BAC-7FE against *S. aureus* ATCC25923 (a) and *B. cereus* MTCC430 (b) determined using the agar well diffusion method with the positive (1 mg/ml streptomycin) and negative control (10% DMSO).

The 7 isolates with high antagonistic activity against *S. aureus* ATCC25923 and *B. cereus* MTCC430 were chosen to determine the bioactive metabolites of their filtrate crude extracts. Two of them (isolates 1BAC-7 and 12BAC-7) exhibited good activity against the tested bacteria with inhibition zone diameters ranging from 8.17 ± 0.29 to 13.00 ± 0.00 mm (Table 4 and Figure 2).

### 3.5. Determination of MIC and MBC of Crude Extracts

The results of MIC and MBC of the crude extracts against *S. aureus* ATCC25923 and *B. cereus* MTCC430 are given in Table 4. Two extracts of note were 1BAC-7FE with a high MIC value (512 *μ*g/ml) which was classified as a moderate inhibitor for *S. aureus* ATCC25923 and *B. cereus* MTCC430, whereas 12BAC-7FE with an MIC value of 256 *μ*g/ml was regarded as a strong inhibitor. It was observed that crude extract from isolate 1BAC-7 had no antibacterial activity with its MBC values being higher than 1,024 *μ*g/ml against *S. aureus* ATCC25923, while 12BAC-7FE had a high value of MBC (512 *μ*g/ml). Furthermore, the extract of isolate 12BAC-7 had a lower MBC value (256 *μ*g/ml) than 1BAC-7FE against *B*. *cereus* MTCC430. The standard antibiotic streptomycin had MIC and MBC values of 3.13 and 6.25 *μ*g/ml, respectively, against the tested pathogenic bacteria.

### 3.6. Molecular Identification and Sequencing of the *16S rRNA* Gene

The partial DNA sequences amplified from presumptive *Streptomyces* isolates 7BAC-1, 11BAC-7, 2BAC-10, 4TAK-5, 9TAK-5, 1BAC-7, and 12BAC-7 were analyzed using a BlastN search. The results indicated that these isolates were identified as *Streptomyces* spp. with 99.3–100% similarity and their GenBank accession numbers were recorded as MZ389213, MZ389214, MZ389215, MZ389216, MZ389217, MT373689, and MT373690, respectively.

## 4. Discussion

A great effort to identify new bioactive substances is necessary because of their advantages in the treatment of currently infectious diseases resulting from multidrug-resistant pathogens. Streptomycetes belonging to the family *Actinobacteria* are well known worldwide for the important production of broad-spectrum secondary metabolites [30, 31]. In the present study, 30 soil samples taken from rice fields in 3 districts of Narathiwat province were investigated for the presence of *Actinomycetes.* The prevalence of 136 *Actinomyces* isolates was the greatest at Tak Bai (41.91%) followed by Bacho (33.09%) and Yi-ngo (25%) districts. This is the first report exploring soil *Actinomycetes* in the southernmost province of Thailand. A previous study conducted in Thailand detected 50 *Actinomyces* isolates from 6 soil samples collected from Chonburi province [32]. In another study, 123 isolates of *Actinomycetes* were found from 37 forest soil samples in Nakhon Ratchasima province, Thailand [33]. *Actinomycetes* are widespread in soils rich in organic matter and can grow on the surface layer of the soil at depths of 5–10 cm [34]. Our results proved that all isolates of *Actinomycetes* were widely distributed in acidic soil with pH between 3.7 and 5.2, and moderate moisture range from 15.5% to 37.9% which corresponds with the previous reports [35–37]. Assorted factors affecting the population density of *Actinomycetes* in soil samples include pH, temperature, and soil water content [8]. The current findings based on the screening of antibacterial activity identified that 19 isolates (13.97%) primarily exhibited encouraging antibacterial activity toward the studied pathogenic bacteria. Most of the active isolates could strongly inhibit the growth of *S. aureus* ATCC25923 and *B. cereus* MTCC430, whereas some *Actinomyces* isolates had weak or moderate activity against *E. coli* ATCC25922 and *S. enterica* serovar Typhimurium ATCC14028. However, *P. aeruginosa* ATCC27853 was the most insensitive bacteria. Sapkota et al. [38] reported that 44.8% of the isolated *Streptomyces* spp. had activity against *S. aureus*, and 10.3% had activity against *E. coli* and *K. pneumonia*, with none having activity against *P. aeruginosa*. These results indicated the dissimilar sensitivity between Gram-positive and Gram-negative bacteria due to the distinctive structural components of their cell walls. Gram-positive bacteria have merely the peptidoglycan layer that does not prevent the penetration of bioactive compounds. On the other hand, Gram-negative bacteria have an outer membrane composed of lipopolysaccharide constituents which provide an efficient blockade to lipophilic solutes and contribute to greater resistance to those compounds [39]. Considering the morphological and biochemical characteristics of the 19 active isolates of *Actinomyces* in the current study, their typical features were in accordance with a member of the genus *Streptomyces*. Thus, all isolates were presumptively identified as *Streptomyces* isolates. Moreover, the gray color of the aerial mycelium was the most predominant (47.37%) followed by white series (31.58%). This was consistent with previous findings that reported gray and white color series were a principal group isolated from soil [40, 41]. Among the active isolates, 7 selected isolates that showed strong inhibitory effects against *B. cereus* MTCC430 and *S. aureus* ATCC25923 were subsequently evaluated for their capability to secrete diffusible bioactive metabolites of filtrate crude extracts based on agar well diffusion assay. The results demonstrated that the antagonistic effects of the crude extracts 12BAC-7FE and 1BAC-7FE against *S. aureus* ATCC25923 were significantly lower than standard streptomycin. However, the mean of inhibition zone of 12BAC-7FE against *B. cereus* MTCC430 was not significantly different to the positive control. The remaining 5 extracts did not have any zones of inhibition against the tested pathogenic bacteria, which contrasted with their antibacterial activity on the agar plate obtained during the primary screening. The results were in agreement with Abussaud et al. [40] that found only 5 of 8 active isolates having activity on agar medium produced antagonistic effects in broth culture. These results may be attributed to differences in the culture media composition, inoculum size, or growth conditions that influence the ability of *Streptomyces* isolates to secrete different active substances on solid growth surfaces and liquid media [8, 42]. Another possible explanation is that some active metabolites discharged by streptomycetes may be destroyed or chemically altered during the solvent extraction process [8, 42]. The extraction of the culture filtrate derived from *Streptomyces* strains using ethyl acetate was utilized broadly to regain powerful secondary metabolites. Lim et al. [43] reported the strongest anti-MDR bacterial activity was from ethyl acetate extracts attained from soil *Streptomyces*. In addition, the ethyl acetate extracts of *Streptomyces anulatus* NEAE-94 had the highest inhibitory effects against multidrug-resistant *S. aureus* compared to other solvent extractions [44]. In the current study, the inhibitory activity of isolate 12BAC-7 against *S. aureus* ATCC25923 and *B. cereus* MTCC430 was evidently stronger than the ethyl acetate crude extract acquired from the isolate 1BAC-7; nevertheless, these 2 active isolates were clearly proven to be *Streptomyces* species with high similarity on the basis of *16S rRNA* gene sequence analysis. The comparison of our findings to the results of an earlier study reveals that the crude ethyl acetate extract of *Streptomyces* sp. FEAI-1 showed a notably higher degree of inhibition against both *S. aureus* and *B. cereus*, with MIC and MBC values comprised in the range of 15.6–125 *μ*g/ml and 62.5–250 *μ*g/ml, respectively [45]. It might be due to the difference of the cultural condition used in the experiments which had an effect on the production of active metabolites. Wang et al. [46] reported diverse parameters of process such as temperature, incubation time, carbon, and nitrogen sources that exert an influence on the secondary metabolites production. Further studies on the optimization of growing conditions and nutritional requirements of both 12BAC-7 and 1BAC-7 isolate are needed for improving the antibacterial metabolite production. In addition, the metabolite crude extracts should also be structurally characterized by means of HPLC-MS to identify the active compounds that may be useful for future development into novel therapeutic agents.

## 5. Conclusion

Our study focused on searching for new, useful bioactive metabolites from soil-derived *Actinomycetes*. The 2 potent *Streptomyces* spp. (isolates 1BAC-7 and 12BAC-7) were isolated from 30 different paddy soils collected in 3 areas of Narathiwat province, Southern Thailand. Although, the ethyl acetate crude extracts formed by both isolates had moderate to strong inhibition against Gram-positive *S. aureus* ATCC25923 and *B. cereus* MTCC430, an isolate of *Streptomyces* sp. (12BAC-7) should be considered as a promising source of effective metabolites against the selected test pathogens.

## Figures and Tables

**Table 1:** Physicochemical characteristics and abundance of *Actinomycete* isolates found in soil samples at diverse sampling areas.

Soil samples^a^	Parameters				
	Texture	Color	pH range	Moisture content (%) range	No. of *Actinomycete* isolates (%)
BAC-1 to BAC-10	Loam, clay loam	Brown	3.7–4.1	15.5–28.1	45 (33.09)
TAK-1 to TAK-10	Clay	Gray, brown	4.6–5.2	23.6–37.9	57 (41.91)
YIN-1 to YIN-10	Loam	Brown	4.0–5.2	16.8–24.5	34 (25)

^a^The soil samples were named with sampling sites and the number of paddy fields. BAC: Bacho; TAK: Tak Bai; YIN: Yi-ngo.

**Table 2:** Primary antibacterial screening of 19 potent *Actinomyces* isolates against 5 pathogenic bacteria using the cross-streak method.

No.	Isolates^a^	Degree of inhibition against tested bacterial pathogens^b^				
		EC	ST	PA	SA	BC
1	7BAC-1	−	−	−	+++	+++
2	11BAC-1	−	−	−	+++	+
3	5BAC-6	−	−	−	+++	+
4	1BAC-7	−	−	−	+++	+++
5	11BAC-7	−	+	−	+++	+++
6	12BAC-7	−	−	−	+++	+++
7	16BAC-7	−	−	−	+	+
8	9BAC-9	−	−	−	+	+
9	2BAC-10	−	+	−	+++	+++
10	9TAK-1	−	−	−	+++	+++
11	1TAK-3	+	−	−	−	+
12	4TAK-5	++	−	−	+++	+++
13	9TAK-5	−	−	−	+++	+++
14	11TAK-6	++	−	−	−	−
15	1TAK-8	++	−	−	−	−
16	2TAK-8	+	−	−	−	++
17	1YIN-1	+	−	−	−	−
18	4YIN-2	+	−	−	−	−
19	5YIN-2	++	−	−	−	−
	Total	8	2	0	12	14

^a^The code name of isolates was designated as the number of isolates found in each soil sample. ^b^EC: *E. coli* ATCC25922; ST: *S. enterica* serovar Typhimurium ATCC14028; PA: *P. aeruginosa* ATCC27853; BC: *B. cereus* MTCC430; SA: *S. aureus* ATCC25923; +++: strong activity; ++: moderate activity; +: weak activity; −: no activity.

**Table 3:** Morphological features and biochemical characteristics of presumptive *Streptomyces* isolates.

Isolates	Morphological and cultural characteristics		Biochemical test^a^							
	Aerial mass color	Spore arrangement	Gram stain	Catalase reaction	Indole production	Citrate degradation	H_2_S production	Urease hydrolysis	Motility test	LDC test
7BAC-1	White	Spiral	+	+	−	+	−	+	−	−
11BAC-1	Gray	Rectiflexibile	+	+	−	−	−	−	−	−
5BAC-6	Gray	Rectiflexibile	+	+	−	−	−	−	−	−
1BAC-7	Gray	Spiral	+	+	−	+	−	+	−	−
11BAC-7	Gray	Spiral	+	+	−	+	−	+	−	−
12BAC-7	Gray	Spiral	+	+	−	+	−	+	−	−
16BAC-7	Gray	Spiral	+	+	−	+	−	+	−	−
9BAC-9	Gray	Retinaculiaperti	+	+	−	+	−	+	−	−
2BAC-10	Gray	Retinaculiaperti	+	+	−	−	−	+	−	−
9TAK-1	White	Retinaculiaperti	+	+	−	−	−	−	−	−
1TAK-3	White	Spiral	+	+	−	−	−	−	−	−
4TAK-5	White	Retinaculiaperti	+	+	−	+	−	+	−	−
9TAK-5	White	Rectiflexibile	+	+	−	+	−	+	−	−
11TAK-6	Yellow	Retinaculiaperti	+	+	−	−	−	+	−	−
1TAK-8	Gray	Retinaculiaperti	+	+	−	+	−	+	−	−
2TAK-8	Orange	Retinaculiaperti	+	+	−	−	−	+	−	−
1YIN-1	White	Retinaculiaperti	+	+	−	−	−	−	−	−
4YIN-2	Yellow	Rectiflexibile	+	+	−	−	−	−	−	−
5YIN-2	Yellow	Spiral	+	+	−	−	−	−	−	−

^a^+: positive; −: negative; LDC test: lysine decarboxylase test.

**Table 4:** Antibacterial property of crude extracts against selected pathogenic bacteria determined using the agar well diffusion assay and colorimetric broth microdilution method.

Crude extract and control	Diameter of the inhibition zone (mm)		MIC/MBC (*μ*g/ml)	
	SA	BC	SA	BC
7BAC-1FE	0.00 ± 0.00^c^	0.00 ± 0.00^c^	nd	nd
1BAC-7FE	8.17 ± 0.29^a^	9.33 ± 1.15^a^	512/>1024	512/512
11BAC-7FE	0.00 ± 0.00^c^	0.00 ± 0.00^c^	nd	nd
12BAC-7FE	8.83 ± 0.29^a^	13.00 ± 0.00^ab^	256/512	256/256
2BAC-10FE	0.00 ± 0.00^c^	0.00 ± 0.00^c^	nd	nd
4TAK-5FE	0.00 ± 0.00^c^	0.00 ± 0.00^c^	nd	nd
9TAK-5FE	0.00 ± 0.00^c^	0.00 ± 0.00^c^	nd	nd
Streptomycin	19.50 ± 0.87^b^	26.50 ± 3.04^b^	3.13/6.25	3.13/6.25

Data are expressed as mean ± SD of 3 replicates. Results with different lowercase superscripts in the same column are significantly different at *P* < 0.05 (Dunnett' T3 test). FE: filtrate of the ethyl acetate crude extract; SA: *S. aureus* ATCC25923; BC: *B. cereus* MTCC430; nd: not determined.

## Data Availability

The data used in this study are included within the article, and the sequence data are available from the NCBI nucleotide database.
